# A Dispensatory and Therapeutical Remembrancer; Comprising the Entire Lists of Materia Medica, Preparations and Compounds, with a Full and Distinct Version of Every Practical Formula, as Authorized by the London, Edinburgh, and Dublin Royal College of Physicians, in the Latest Editions of Their Several Pharmacopœias; to Which Are Subjoined Copious Relative Tables, Exemplifying Approved Forms under Which Compatible Medicines, &c., May Be Extemporaneously Combined, &c. &c. &c.

**Published:** 1848-05

**Authors:** 


*A Dispensatory and Therapeutical Remembrancer; comprising the entire lists of Materia Medica, Preparations and Compounds, with a full and distinct version of every practical Formula, as authorized by the London, Edinburgh, and Dublin Royal College of Physicians, in the latest editions of their several Pharmacopœias; to which are subjoined copious relative tables, exemplifying approved forms under which compatible medicines, &c., may be extemporaneously combined, &c. &c. &c.* By John Mayne, M. D., L. R. C. S., Edinburgh. *Revised, with the addition of the formulæ of the United States Pharmacopœia, &c.* By R. Eglesfeld Griffith, M. D., Author of "Medical Botany," &c. &c. 12mo. pp. 329. Lea & Blanchard. Philadelphia: 1848.

This Dispensatory is intended by its author as "an unabridged *practical* formulary of the three British Pharmacopœias; and this in addition to a full amount of collective information as to the uses, &c., of the different medicines, and other important points relating to remedial means and appliances." "Another feature of originality"—the author adds—"which, it is expected, will prove highly serviceable, is the introduction—wherever deemed requisite—of extemporaneous formulæ into the work. These are separated from the pharmacopœial or continuous text of each page where they occur, by a short horizontal line, in the manner of foot-notes; and, it need scarcely be explained, are intended to assist the practitioner's memory, by suggestion of forms and combinations most suitable for the medicinal substances to which they are annexed." p. vi.

The preparation of the American edition has been confided to one every way competent to the task, and, so far as we have had an opportunity for examination, has been well accomplished. We do not know, indeed, that we could do better than endorse the statement of Dr. Griffith as contained in the "Preface to the American edition." "This work," he says, "is so excellent a compend of the officinal processes directed by the three British Colleges, and in so convenient and portable a form, that an edition of it, also comprising those of the United States Pharmacopœia, cannot fail to be useful to the medical profession in this country. In revising it for publication, great care has been taken to supply deficiencies and to correct any errors. The additions have been very copious, including the formulæ of the U. S. Pharmacopœia, and some from other sources. The English names of the various preparations have also been appended, and a double index of these and the Latin appellations given. The work now gives a clear and comparative view of all the officinal preparations directed by the authorities referred to, so as to enable the prescriber to see at a glance wherein they differ, and to select such as are best suited to fulfil the indication of the case."

